# Glycerol photocatalytic oxidation to higher value-added compounds via bismuth oxyhalide photocatalysts

**DOI:** 10.1038/s41598-023-42246-3

**Published:** 2023-09-11

**Authors:** Paphada Limpachanangkul, Prathana Nimmmanterdwong, Licheng Liu, Mali Hunsom, Kejvalee Pruksathorn, Pornpote Piumsomboon, Benjapon Chalermsinsuwan

**Affiliations:** 1 Fuels Research Center, Department of Chemical Technology, Faculty of Science, Chulalongkorn University, Pathumwan, Bangkok, 10330 Thailand; 2 Department of Chemical Engineering, Faculty of Engineering, Mahidol University, Salaya, Nakhon Pathom, 73170 Thailand; 3 Key Laboratory of Marine Chemistry Theory and Technology (Ministry of Education), College of Chemistry and Chemical Engineering, Ocean University of China, Qingdao, 266100 Shandong China; 4 Center of Excellence On Petrochemical and Materials Technology, Chulalongkorn University, Pathumwan, Bangkok, 10330 Thailand; 5 Advanced Computational Fluid Dynamics Research Unit, Chulalongkorn University, Pathumwan, Bangkok, 10330 Thailand

**Keywords:** Engineering, Nanoscience and technology

## Abstract

Bismuth oxyhalides (BiOX) including BiOCl, BiOBr, and BiOI, were well synthesized using solvothermal technique and then used in the aqueous phase photooxidation of glycerol as a catalyst. The as-synthesized BiOBr could achieve the highest glycerol transformation of around 85.6% in 8 h under ultraviolet light (UV) irradiation among as-synthesized BiOXs. Moreover, the BiOBr/TiO_2_ heterojunction was also prepared through an ethylene glycol-assisted solvothermal process. This new BiOBr/TiO_2_ heterostructure exhibited excellent photocatalytic activity (97.4%) for the oxidation of glycerol compared with pure BiOBr (74%) under ultraviolet light irradiation at 6 h. This obtained behavior was confirmed by more produced OH^•^ radicals of BiOBr/TiO_2_.

## Introduction

Due to the increase in environmental consciousness throughout the world, alternative fuels produced from renewable biomass resources have become a globally high attraction ^1,2^. Biodiesel is one of the most commonly used biofuels because it can replace petroleum-derived diesel and has much lower levels of harmful emissions ^3^. Many years ago the enormous growth of the biodiesel industry resulted in oversupplied of the existing demand for glycerol which is the by-product of the transesterification process ^4–6^. Because of the unique presence of three hydroxyl groups in glycerol structure, this aqueous glycerol molecule has been used as a desirable reactant for converting into other key compounds to avoid its low price issue ^7,8^.

The oxidation of glycerol into high-added-value compounds has been a research avenue that attracts special attention. A variety of possible generated products such as dihydroxyacetone, glyceraldehyde, glyceric acid, tartronic acid, and glycolic acid can be obtained from a multitude of reaction pathways ^9,10^. Nevertheless, the major challenges associated with the glycerol oxidation process at a commercial scale are (i) an alkaline condition necessity, (ii) high temperature and pressure operations, and (iii) the deactivation of the utilized catalyst ^11^. To address these problems, new processes with green energy technologies have been considered.

The heterogeneous photocatalytic process has received much attention during the last three decades as a consequence of its advantages including ambient temperature and pressure operation, high process efficiency, environmentally friendly nature, complete mineralization of parents and their intermediate compounds without leaving secondary pollution, and cost-effective ^12–14^. Therefore, photocatalysis has been employed in many literature studies and becoming a dependable technological approach in many industries for a wide range of promising environmental applications ^15,16^.

Nowadays, semiconductor metal oxides with different band gap energies and oxidizing powers are usually used as photocatalysts such as TiO_2_, ZnO, Fe_2_O_3_, SiC, CdS, and WO_3_. Interestingly, bismuth oxyhalides (BiOX; X = Cl, Br, I) have been utilized in various applications including selective oxidation of alcohols, organic synthesis, water splitting, indoor-gas purification, and photodegradation of organic pollutants in wastewater ^17–20^. These materials are indirect band gap semiconductors composed of [Bi_2_O_2_] slabs interleaved by double slabs of halogen atoms resulting in their complex band structures and unique layered structures which enables it attractive for anisotropic structural, electrical, optical, mechanical, and catalytic properties ^21,22^.

BiOX exhibits indirect band gap nature. The band gap of BiOX decreases as the atomic number of X element increases which makes their response wavelengths gradually move towards the visible light region from UV light region. BiOCl with a wide band gap of 3.2 eV is active only in the UV light region, while BiOBr and BiOI have appropriate band gaps of 2.6 and 1.8–2.1 eV, respectively, which results in efficient utilization of solar spectrum in visible light ^23^.

The determination of the valence band edge of BiOX is important elementary property to understand the redox power of photogenerated carriers. The semiconductor with higher valence band maximum can produce relatively more generated oxidation holes providing strong photocatalytic oxidation ability. Simultaneously, more electrons are produced in the up-shifting of conduction band minimum which then reduce more electron acceptor species ^24^. Therefore, the incorporation of chloride, bromide and iodide ions within the nanosized BiOX samples, respectively, greatly increase conduction band minimum and decrease valence band maximum position resulting in modulating the photocatalytic oxidation power ^25^.

Additionally, the photocatalytic behavior of semiconductor photocatalysts, including BiOX, depend on many factors, such as crystalline, morphology, surface area, preparation methods, starting materials and organic structures, etc. Among these factors, halide anions largely influence the photocatalytic efficiency of BiOX due to its different electronegativity and ionic radius. In addition, the photocatalytic performance of several photocatalysts is affected by a number of operating factors, such as amount of photocatalyst, illumination source, type and concentration of pollutant used, synthesis method, pH and temperature, etc. However, their structural, surface morphological and optical properties, which is controlled by the synthesis methods, play an important role in the determining the photocatalytic reactivity ^26^.

Generally, liquid-phase synthesis methods, including hydrothermal and solvothermal synthesis, reversed-phase micro-emulsion method, interface-mediated synthesis, sonochemical method, templated synthesis and anion-exchange synthesis, are frequently used methods for synthesizing BiOX with the desired functionality. Out of all these, solvothermal method is one of the simplest and most convenient methods for the synthesis of BiOX. Its high temperature and pressure conditions could lead to differences in size, shape, and crystallinity and in some cases, non-stoichiometric BiOX ^27^. The advantage of this preparation method is the enhancement of the solubility of the precursors and initiates the formation of bismuth-based coordination compounds. Moreover, the improvement in crystallinity of the synthesized nanostructures is another significant benefit of this method. Because crystallinity governs many physical properties of the pristine nanoparticles such as surface area, surface energy, lattice distortion and light absorptivity, so it is one of the vital parameters that ultimately influences the photocatalytic response.

There is a wide range of diverse solvent with different properties which play a crucial role in determination the morphology of the synthesized material. The most commonly used solvents are water and organic solvents. However, water have narrow temperature range of aqueous media (0–100 °C) which limits its application. Accordingly, organic solvents such as ethanol, methanol, and ethylene glycol were utilized to dissolve both bismuth source and halogen source. The common source for bismuth includes Bi(NO_3_)_3_⋅5H_2_O, Bi(NO_3_)_3_, NaBiO_3_⋅2H_2_O, Bi_2_O_3_, Bi, BiCl_3_, and BiI_3_ while surfactants like hexadecyl trimethylammoniumchloride (CTAC) and hexadecyltrimethylammoniumbromide (CTAB), halogen acids HX, NaX, KX and ionic liquids with halogen element served as halogen source in most cases ^28,29^. Furthermore, the pH value, reaction temperature, and reaction time also have an effect on the synthesized BiOX.

The basic structure of the prepared BiOX catalysts were determined using a variety of characterization methods. For the analysis of the surface morphology and elemental composition, scanning electron microscopy–energy dispersive X-ray spectroscopy (SEM–EDS) was used. The catalysts were determined surface area and phase composition using Brunauer–Emmett–Teller (BET) analysis and X-ray diffraction (XRD), respectively. Moreover, Diffuse reflectance UV–Vis spectroscopy (UV–Vis–DRS) were also utilized to define the optical properties ^30^.

In addition, constructing a built-in electric field at the heterojunction interface in catalysts is considered an effective approach to achieve an effective separation of photogenerated charge carriers to improve the photocatalytic activity of pure photocatalytic materials ^31,32^. The broad range of band redox potential of BiOX ensures that these materials can easily be a good partner with a wide variety of semiconducting materials to form the nanocomposite ^33^. These composite partners form a heterojunction that enables the photogenerated electrons and holes to spatially separate, and thereby reduce the charge recombination probability.

Lee et al. ^29^ synthesized hierarchical BiOXs for the photocatalytic hydrogen evolution from water splitting via the irradiation of visible light. BiOI showed the maximum hydrogen production rate because of its lowest photoluminescence (PL) intensity and sufficient over-potential of the conduction band. Lv et al. ^24^ fabricated BiOX nanosheets to investigate their photocatalytic performance. BiOCl showed the highest photocatalytic oxidation of organic contaminants with different charges, which was mainly ascribed to the lowest valence band maximum edge. Jin et al. ^34^ designed a robust bismuth-rich hollow Bi_4_O_5_Br_2_ microspheres for efficient photocatalytic CO_2_ reduction. Benefiting from both bismuth-rich strategy and hollow structure, the elevated conduction band potential, and large specific area were provided which facilitated the charge transfer and photoreduction ability and enhanced the CO_2_ adsorption and activation. However, the application of BiOXs for photocatalytic oxidation of glycerol is still lacking.

In this study, BiOXs and BiOBr heterojunction with TiO_2_ were both successfully synthesized by a solvothermal method. This study objective was to explore the photocatalytic performance of pure BiOXs and the BiOBr/TiO_2_ heterojunctions for glycerol conversion using UV light irradiation at ambient conditions.

## Experimental

### Chemicals

All the chemicals employed in the experiments were obtained commercially with the analytical grade without further purification, containing bismuth nitrate pentahydrate (Bi(NO_3_)_3_⋅5H_2_O, Sigma-Aldrich), potassium Chloride (KCl, Ajax Finechem), potassium Bromide (KBr, 98%, KemAus), potassium Iodide (KI, 99.0%, Ajax Finechem), ethylene glycol (C_2_H_6_O_2_, KemAus), ethanol absolute (C_2_H_6_O_3_, 99.9%, QReC), hydrogen peroxide (30 wt%, Merck), glyceraldehyde (GCD, 98%, Sigma Aldrich), glyceric acid (GCA, 20 wt%, TCI), glyoxylic acid (C_2_H_2_O_3_, 98%, Sigma Aldrich), glycolic acid (GCOA, 70 wt%, Ajax Finechem), formic acid (FMA, 98%, Merck), dihydroxyacetone (DHA, 98%, Merck) and glycerol (GLY, 99.5%, QReC). The probe compounds used to determine OH^•^ radical levels were *para*‐chlorobenzoic acid (*p*CBA, Sigma Aldrich).

### Photocatalysts preparation^43^

#### Synthesis of BiOXs

BiOXs were prepared using a solvothermal technique. In general preparation, 2 mmol of Bi(NO_3_)_3_⋅5H_2_O and 2 mmol of KX (X = Cl, Br, or I) were separately dispersed in 15 mL ethylene glycol. Then, the two suspensions were blended for 30 min by magnetically stirring. The mixture was then put into a 50-mL Teflon-line autoclave, heated for 12 h at 120 °C. After the autoclave cooled down to room temperature, the obtained precipitants were filtered out, continually rinsed with absolute ethanol and deionized water. Accordingly, the prepared samples were dried for 12 h at 60 °C before being collected.

#### Synthesis of BiOBr/TiO_2_ heterojunction

About 0.32 mmol of TiO_2_ (258 mg), Bi(NO_3_)_3_⋅5H_2_O (156.9 mg), and KBr (38.5 mg) were separately dissolved in ethylene glycol. Bi(NO_3_)_3_ solution was dropped into the white TiO_2_ suspension for 15 min. Afterward, the KBr solution was dropped into the previous mixture gradually. After stirring for 60 min, a yellow solution was put into the Teflon-lined autoclave and kept for 3 h at 120 °C. The obtained precipitates were washed with ethanol and deionized water and dried. The powders were identified as BiOBr/TiO_2_.

### Characterization

The crystallite structure of as-prepared photocatalysts was analyzed by the powder X-ray diffraction (XRD) using a D8 Discover-Bruker AXS X-ray diffractometer at 40 mA and 40 kV equipped with Cu-K*α*.

### Photocatalytic performance evaluation

The as-prepared BiOX samples was evaluated its photocatalytic oxidation of glycerol at room temperature in a cylindrical glass reactor having a total volume of 250 mL under UV light irradiation. For each experiment, the employed photocatalyst (300 mg) was dissolved in the glycerol solution (0.3 mol L^−1^, 100 mL). The suspension was agitated continuously by a magnetic stirrer at 400 rpm for 30 min in the dark to provide a well dispersion and adsorption–desorption equilibrium of glycerol on the photocatalyst surface. Then, approximately 15.32 mL of 30 wt.% H_2_O_2_ was inserted into the mixture before the irradiation. A 120W UV high-pressure mercury lamp (RUV 533 BC, Holland) was employed as a light source at the light intensity of 5.93 mW/cm^2^. An aliquot of 2 mL was then sampled and centrifuged for the following analysis in each time-interval.

The quantitative analysis of glycerol and generated chemical species were characterized by high-performance liquid chromatography (HPLC) with a RID-10A refractive index detector (Shimadzu) during 8 h of reaction time. The stationary phase was the aminex HPX-87H ion exclusion (300 mm × 7.8 mm), and the mobile phase was an acetonitrile–water solution (70:30 V*/V*) with 5 mM H_2_SO_4_ at a constant flow rate of 0.4 mL/min. Standard solutions of glycerol and expected major product compounds were used to define the relationship between the peak area and concentration. The glycerol conversion (*X*) and product selectivity (*S*) obtained from the reaction were computed respectively according to Eqs. (1) and (2):

1 $$X_{GLY} (\% )^{{}} =^{{}} \frac{{{\text{amount}}^{{}}\,{\text{of}}^{{}}\,{\text{glycerol}}^{{}}\,{\text{converted}}}}{{{\text{Total}}^{{}}\,{\text{amount}}^{{}}\,{\text{of}}\,{\text{glycerol}}^{{}}\,{\text{in}}^{{}} {\text{reactant}}}} \times 100\%$$

2 $$S(\% )^{{}} =^{{}} \frac{{{\text{amount}}^{{}}\,{\text{of}}^{{}}\,{\text{desired}}^{{}}\,{\text{product}}\,^{{}} j^{{}}\,{\text{formed }}}}{{{\text{amount}}^{{}}\,{\text{of}}^{{}}\,{\text{all}}^{{}}\,{\text{products}}^{{}}\,{\text{formed}}}} \times 100\%$$

### OH^•^ radical measurement

The formation rate of OH^•^ radical was measured by the reduction of *p*CBA. The content variation of *p*CBA in the samples was analyzed by HPLC instrument using Shimadzu Shim-pack GIST C18 column (250 × 4.6 mm) with 5 μm particle size. The mobile phase consisted of 10 mM H_2_SO_4_ in methanol‐water‐acetonitrile solution (55:35:10 *V*/*V*). The flow rate of the mobile phase was 0.4 mL/min and the injection volume was 10 μL.

## Results

### Crystallite structure analysis^43^

**Figure 1:**
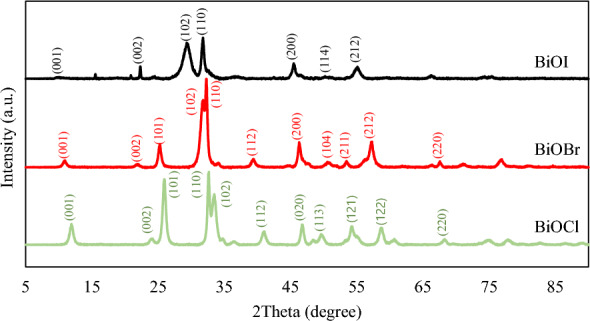
XRD patterns of as-prepared BiOXs^43^.

To investigate the crystalline phase of all the BiOXs, X-ray diffraction patterns were analyzed and shown in Fig. 1. The key pattern of BiOCl at 12.02° (0 0 1), 24.10° (0 0 2), 25.99° (1 0 1), 32.30° (1 1 0), 33.49° (1 0 2), 41.05° (1 1 2), 46.83° (0 2 0), 49.89° (1 1 3), 54.30° (1 2 1), 58.83° (1 2 2) and 68.46° (2 2 0) were similar to the JCPDS file no. 06–0249 ^35^. For BiOBr, its distinctive pattern at 10.83° (0 0 1), 21.95° (0 0 2), 25.26° (1 0 1), 31.77° (1 0 2), 32.30° (1 1 0), 39.38° (1 1 2), 46.25° (2 0 0), 50.78° (1 0 4), 53.39° (2 1 1), 57.15° (2 1 2) and 67.57° (2 2 0) were consistent with the JCPDS Card no. 09–0393 ^28^. The pattern of BiOI at 10.88°, 22.08°, 29.4°, 31.78°, 45.44°, 50.9°, and 55.2°, which belonged to (0 0 1), (0 0 2), (1 0 2), (1 1 0), (2 0 0), (1 1 4) and (2 1 2) crystal planes respectively, were related to the JCPDS file no. 10–0445 ^36^. From the XRD patterns of each sample, the typical diffraction peaks of the tetragonal structure were found in all three prepared BiOX samples. All sample had intense and clear diffraction peaks, and no peaks of impurities were found, which indicated that the samples had a high purity and high degree of crystallinity.

### Photocatalytic performance test for glycerol conversion^43^

**Figure 2:**
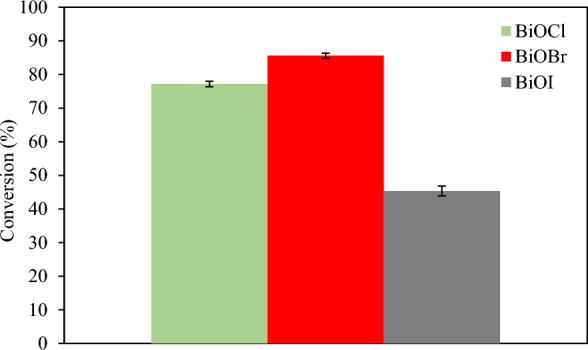
Photocatalytic conversion with various BiOX photocatalysts at 8 h reaction time^43^.

**Figure 3:**
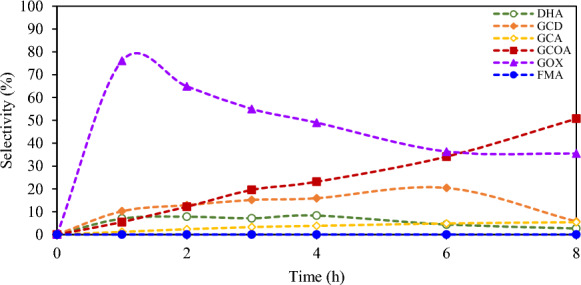
Selectivity of generated compounds from glycerol conversion over BiOBr photocatalyst versus time and their products: dihydroxyacetone (DHA), glyceraldehyde (GCD), glyceric acid (GCA), glyoxylic acid (GOX), glycolic acid (GCOA) and formic acid (FMA)^43^.

The photocatalytic activity of each prepared BiOX with 0.3 M glycerol is shown in Fig. 2. The BiOBr sample exhibited the highest glycerol conversion among other prepared photocatalysts followed by BiOCl and BiOI, respectively. This may be due to a more proper bandgap of 2.64 eV for BiOBr and preferable UV–Visible light photocatalytic activity ^37^. The low glycerol conversion rate of BiOCl and BiOI might be attributed to the fact that unmodified BiOCl only expressed photocatalytic activity under UV light as a result of its widest band gaps of 3.22 eV and pure BiOI had the narrowest bandgap of 1.77 eV resulted in poor photocatalytic activity due to the inherent rapid electron–hole recombination ^38^. GOX was more observed among other generated chemical products with highest selectivity of 76% at the first hour of irradiation time as shown in Fig. 3, while the others, including DHA, GCD, GCA, GCOA and FMA, were obtained less than 11%.

However, the oxidation reaction of water molecules only found in the presence of BiOCl and BiOBr photocatalysts due to the higher redox potential of water molecules than the valence band of BiOI ^39^. The OH^•^ radicals were produced from water oxidation and could further oxidize the glycerol molecule, giving a higher photocatalytic conversion of glycerol.

**Figure 4:**
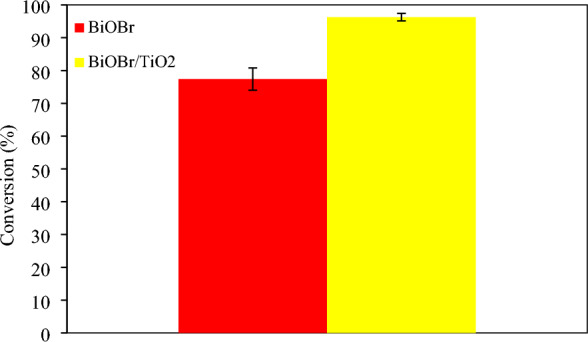
Photocatalytic conversion of BiOBr and BiOBr/TiO_2_ photocatalysts with 0.3 M glycerol at 6 h reaction time^43^.

**Figure 5:**
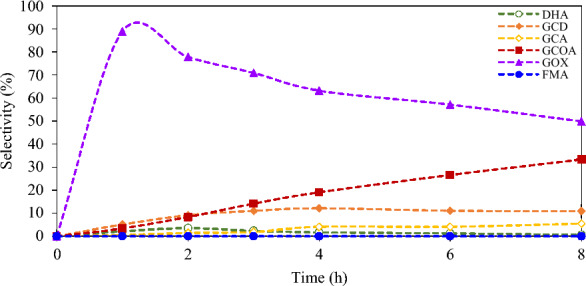
Selectivity of generated compounds from glycerol conversion over BiOBr/TiO_2_ photocatalysts versus time and their products: dihydroxyacetone (DHA), glyceraldehyde (GCD), glyceric acid (GCA), glyoxylic acid (GOX), glycolic acid (GCOA) and formic acid (FMA)^43^.

In this study, the comparison of glycerol conversion between BiOBr and BiOBr/TiO_2_ photocatalysts was also investigated as shown in Fig. 4. It was found that the BiOBr/TiO_2_ photocatalyst had notably higher photocatalytic activity which originated from the unique relative energy band positions of these two semiconductors. When the anatase TiO_2_ and BiOBr semiconductors are in contact, a *p*-*n* heterojunction will be formed, which has a type-II band alignment ^40^. Consequently, the absorption of visible light can be expanded by BiOBr, resulting in promoting the transfer of charge carriers and inhibiting the recombination of photoexcited electrons and holes ^41^. Regarding the generated products, GOX was also more produced among other generated chemical products as shown in Fig. 5.

During the photocatalytic process, the electrons (e^−^) are generated at the valence band (VB) of BiOBr and translated to its conduction band (CB), and the photo-generated holes (h^+^) will transfer from the valence band of BiOBr to the valence band of anatase TiO_2_ by the formed interfacial heterojunctions. In addition, the excited electrons will also migrate from the conduction band of anatase TiO_2_ to BiOBr, which can separate and transfer the photo-generated electron–hole pairs ^42^. The photoinduced holes at the valence band of anatase TiO_2_ can make the glycerol adsorbed on the photocatalyst surface to be oxidized. The photogenerated electrons then react with the dissolved oxygen molecules to form the main reactive species ˙O^2−^ and then further oxidize the glycerol molecule. Moreover, band gaps value of BiOBr/TiO_2_ may decrease compared to bare BiOBr which then promotes the photo-generated carriers.

The routes of GOX formation were previously explored. GOX can be directly produced from the C–C cleavage of glycerol, DHA, GCD and GCA molecules. Briefly, the 1°-C and 2°-C atom of glycerol can be easily attached by an excess HO^•^ radicals to form GCD and DHA, respectively. The generated GCD can be further oxidized to GCA via the HO^•^ radicals. OH^•^ radical and free O_2_ can selectively attach the C atom of DHA, GCD and GCA yielding the hydroxypyruvaldehyde (C_3_H_4_O_3_) as intermediate compound. This generated intermediate species was sequentially attached by OH• radicals resulting in the cleavage of its C–C bond to form the final product, GOX and methanediol (CH_4_O_2_) ^43^. Moreover, OH^•^ radicals can also attach and cleave the C–C bond of GCA, resulting in the formation of GCOA and a trace of FMA. Simultaneously, the DHA can also be oxidized to GCOA.

### OH^•^ radical measurement

**Figure 6:**
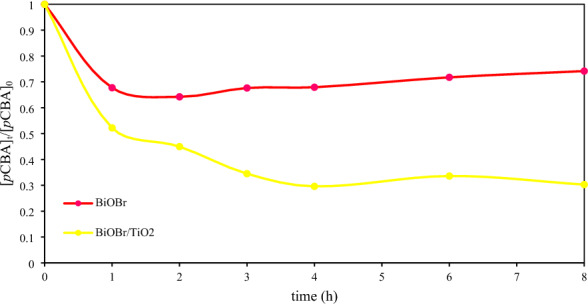
Variation of *p*CBA content as a function of irradiation time.

To prove that OH^•^ radical was largely produced in the BiOBr/TiO_2_ system than BiOBr system, an additional experiment was carried out. As demonstrated in Fig. 6, the degradation profile revealed that more *p*CBA content was eliminated in the BiOBr/TiO_2_ system than BiOBr system. The BiOBr/TiO_2_ heterojunction led to enhanced generation of OH^•^ radicals. Therefore, more glycerol molecules were attached and further oxidized to form the final product as previously stated in the reaction mechanisms of glycerol conversion. However, both BiOBr and BiOBr/TiO_2_ systems mostly reduced OH^•^ radicals in the first 4 h.

## Conclusions

BiOX photocatalysts were well produced with a solvothermal technique. The as-synthesized photocatalysts were examined its photocatalytic activities by monitoring the glycerol and photogenerated product concentrations. BiOBr can achieve the highest glycerol conversion in 8 h under UV light irradiation compared to other BiOXs. Moreover, BiOBr/TiO_2_ was also fabricated by an ethylene glycol-assisted solvothermal process. The prepared BiOBr/TiO_2_ heterojunctions displayed more excellent performance for the glycerol transformation than pure BiOBr which is benefiting from energy band structure by promoting the transfer of charge carriers and inhibiting the photoexcited electrons and holes’s recombination. This obtained behavior was confirmed by more produced OH^•^ radicals of BiOBr/TiO_2_. In the future, the data on the photocatalytic activity of BiOXs and BiOXs heterojunction for glycerol oxidation under visible light irradiation should be further investigated.

## Data Availability

The datasets used and/or analysed during the current study available from the corresponding author on reasonable request.
